# Exploring Adversarial Robustness of LiDAR Semantic Segmentation in Autonomous Driving

**DOI:** 10.3390/s23239579

**Published:** 2023-12-02

**Authors:** K. T. Yasas Mahima, Asanka Perera, Sreenatha Anavatti, Matt Garratt

**Affiliations:** 1 School of Engineering and Technology, University of New South Wales, Canberra, ACT 2612, Australia; yasas.mahima@adfa.edu.au (K.T.Y.M.); agsrenat@adfa.edu.au (S.A.); m.garratt@adfa.edu.au (M.G.); 2 School of Engineering, University of Southern Queensland, Brisbane, QLD 4300, Australia

**Keywords:** adversarial attacks, LiDAR, semantic segmentation, autonomous vehicles

## Abstract

Deep learning networks have demonstrated outstanding performance in 2D and 3D vision tasks. However, recent research demonstrated that these networks result in failures when imperceptible perturbations are added to the input known as adversarial attacks. This phenomenon has recently received increased interest in the field of autonomous vehicles and has been extensively researched on 2D image-based perception tasks and 3D object detection. However, the adversarial robustness of 3D LiDAR semantic segmentation in autonomous vehicles is a relatively unexplored topic. This study expands the adversarial examples to LiDAR-based 3D semantic segmentation. We developed and analyzed three LiDAR point-based adversarial attack methods on different networks developed on the SemanticKITTI dataset. The findings illustrate that the Cylinder3D network has the highest adversarial susceptibility to the analyzed attacks. We investigated how the class-wise point distribution influences the adversarial robustness of each class in the SemanticKITTI dataset and discovered that ground-level points are extremely vulnerable to point perturbation attacks. Further, the transferability of each attack strategy was assessed, and we found that networks relying on point data representation demonstrate a notable level of resistance. Our findings will enable future research in developing more complex and specific adversarial attacks against LiDAR segmentation and countermeasures against adversarial attacks.

## 1. Introduction

With the rapid development of Artificial Intelligence (AI), Deep Learning (DL) networks have become the state-of-the-art technology for a wide range of computer vision tasks. With the availability of large datasets, at present, these DL networks are used to perform object identification, object tracking, etc., tasks in safety-critical applications [1]. Autonomous vehicles (AVs), in particular, are a promising component of smart cities that rely on various DL networks to monitor the surrounding environment and transit safely. Globally, there are several AV-related initiatives to develop fully automated vehicles and nowadays there are highly automated vehicles in public services such as Google Waymo [2].

The initial iteration of AVs featured perception systems that relied on DL networks based on 2D camera images. However, due to the complex environment of autonomous driving, the commercial and scientific level AVs gradually migrated to employ 3D perception technologies. In order to perform 3D perception tasks, sensors such as Light Detection and Ranging (LiDAR), and stereo cameras along with complex deep learning architectures are heavily used, as they enable AVs to identify depth information about the scene [3,4,5,6].

Despite the exceptional performance of DL networks, recent research beginning with [7,8] has demonstrated that they are extremely vulnerable to adversarially designed inputs (known as adversarial attacks) that are usually visually identical to the original input and are intended to deceive the network’s prediction. Initially, adversarial attacks were mainly investigated in the computer vision domain. However, a significant amount of research has been since been conducted on adversarial attacks in order to identify vulnerabilities of networks based on other input types such as texts, graphs, etc. [9]. The susceptibility of DL networks to adversarial attacks raises concerns regarding their use in safety-critical applications like AVs, as the security of AVs is correlated with the DL networks those AVs employ. As a result, adversarial attacks against AVs have attracted a lot of attention, and numerous studies were conducted to examine the adversarial vulnerabilities of AVs and defend against them [10,11].

Previous studies on adversarial attacks and defense methods against AV perception tasks mainly focused on 2D image-based object recognition [12,13,14,15], and steering networks [16]. Later, researchers extended these investigations to LiDAR-based 3D object detection [17,18,19,20]. To deceive image-based perception methods and 3D object detection, existing adversarial attacks have used noise perturbation-based techniques [12,16] or, to improve physical realizability, have used adversarial objects [17,18] and patch [13,14,15]-based techniques. Nevertheless, the adversarial robustness of LiDAR-based 3D semantic segmentation has not been sufficiently explored.

**Figure 1:**
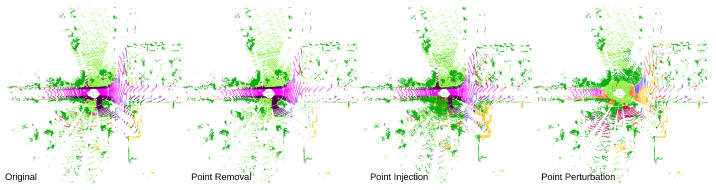
Results from adversarial Attacks. The left-hand side image shows the network’s segmentation results for clean input. The second image demonstrates the segmentation results after the point removal attacks, while the third image shows the results after the point injection attack. The final image shows the segmentation results under the point perturbation attack.

In this study, three LiDAR point-based adversarial attack methods against semantic segmentation networks are assessed. LiDAR semantic segmentation is the primary concentration of this study, as it is more complex than the approximate region-based 3D object detection and point cloud classification methods. In particular, we investigate point removal, point attachment, and point perturbation attacks (See Figure 1) on six different LiDAR semantic segmentation networks developed on the SemanticKITTI dataset [21] covering networks based on points, voxels, and point-voxel data representation strategies. Then, the effect of point distribution on adversarial robustness is investigated. Further, the imperceptibility of the attack methods at various severity levels is evaluated. Following that, the transferability of the attack methods is analyzed in a black-box manner. To the best of our knowledge, this is the first comprehensive investigation of the adversarial robustness of LiDAR semantic segmentation against previously mentioned point-based attack methods. The main contributions of our study are as follows:

1. We update and develop point removal, point attachment, and point perturbation attacks against six LiDAR segmentation networks and also examine how these attack techniques can be applied across different networks.
2. Specifically, a dual loss function-based optimization process is employed for norm-bounded iterative perturbation attack methods to regulate the imperceptibility and is benchmarked against the l2 norm-bounded attacks.
3. A novel evaluation metric is introduced to measure the impact on the original point cloud after the adversarial point injection and removal attacks.
4. We analyze the adversarial sensitivity of each class and the impact of the class-wise point distribution towards the adversarial robustness.

The remainder of the paper is structured as follows: Section 2 summarises the state-of-the-art works. Section 3 discusses the adversarial example generation mechanisms used in our study. Evaluation metrics used to assess the adversarial robustness and attack imperceptibility are presented in Section 4. Section 5 summarises our experimental setup, including the network architectures and dataset. In Section 6, we present the results of the attacks under different severity levels. Section 7 focuses on the evaluation of attack imperceptibility. Section 8 presents an analysis of the cross-network transferability of the attack methods. There, the impact of the sparse tensor quantization pre-processing step towards adversarial robustness is further evaluated. Section 9 discusses our findings and potential research directions. Finally, Section 10 concludes the paper.

## 2. Related Works

### 2.1. Deep Learning for LiDAR Segmentation

Deep neural networks demonstrate high accuracy in image-based object detection and segmentation tasks. Grounded in these networks, researchers introduce DL networks to segment the LiDAR point clouds. Based on the data representation strategy, these networks could be divided into four main categories: point, voxel, point-voxel, and projection-based networks [22]. Point-based networks learn the geometric information from the raw point clouds, while voxel-based networks transform point clouds to compact volumetric grids. Generally, the voxel-based methods enable competitive performance while using less computational resources. Projection-based methods transform the point cloud onto a 2D image and make use of 2D convolution operators to provide the predictions. However, the projection-based methods’ performance is limited by occlusions and scale issues.

### 2.2. Adversarial Attacks against 3D Perception

Adversarial attacks against image-based 2D driving scene segmentation and object recognition have been studied extensively. However, as AVs increasingly leverage 3D perception, there has been a growing focus on studying the adversarial vulnerabilities of 3D perception tasks. The 3D attack methodologies on LiDAR point clouds primarily centre around manipulating the LiDAR point clouds, such as by changing the geometry of the objects via LiDAR point shifting, and adversarial objects. In contrast, 2D attacks aim to compromise networks relying on camera inputs through pixel-level manipulations such as adding noise and adversarial patches. Notably, 2D image-based networks exhibit a higher vulnerability to imperceptible adversarial perturbations, while 3D LiDAR point cloud-based networks demand more substantial manipulations to alter the predictions. The main reason for this is that LiDAR sensing enables the acquisition of comprehensive depth information and allows the DL networks to learn geometry or both geometry and texture information whereas 2D image-based networks mainly rely on texture information [22,23].

In the realm of adversarial attacks on AVs’ 3D perception, a considerable amount of studies are focused on approximate region-based 3D object detection networks based on LiDAR point clouds, camera and LiDAR fusions, and monocular/stereo vision. These attack methods mostly rely on point injection techniques along with adversarial optimization methods [19]. In contrast, another set of studies proposed physically realizable attack methods using adversarially optimized mesh objects [24,25]. These adversarial mesh object-based attacks have proven their success in altering the performance of both LiDAR-based and Multi-Sensor-Fusion (MSF) based networks. Moreover, a limited number of studies have investigated adversarial noise perturbation and patch attacks against camera image-based 3D object detection [26].

The adversarial robustness of LiDAR segmentation of AVs is a relatively unexplored topic. Zhu et al. introduced a real-world object-based adversarial attack against LiDAR segmentation [27]. The fundamental concept behind this study is to determine the most optimal locations at which to place the adversarial point clusters in order to deceive the network and then place real-world objects in those places. However, prior to performing the attack, the adversary has to gather the location’s point cloud to determine the most optimal place to position the adversarial objects. Xu et al. presented an adversarial perturbation-based attack against point cloud segmentation with the intention of degrading the performance and hiding objects [28]. They first demonstrated that color features are more vulnerable than the point coordinates, and conducted their experiments on perturbing the color features. Their evaluations were carried out based on the perception of delivery robots, and for the experiments, they used only the point-based segmentation networks. In [17], Chen et al. experimented with a physically realizable attack against LiDAR segmentation networks available in Baidu Apollo using 3D printable adversarial mesh objects. Moreover, Christian et al. developed a realistic test scenario generation method for LiDAR segmentation using mutations such as object removal, addition, and performing transformation on objects [29]. However, this method cannot be upgraded as an adversarial attack, because adding or removing a complete object digitally makes a significant change to the original point cloud and makes it suspicious to humans.

The study in [30] shares similarities with our study, in which the authors evaluated the three adversarial attacks focused on in our study against different 3D object detection networks. Moreover, Ref. [31] presented a comprehensive analysis of image semantic segmentation against pixel perturbation attacks. However, our study focuses on the adversarial robustness of 3D LiDAR semantic segmentation networks. We present an optimization guided by dual loss functions for iterative norm-bounded perturbation attacks and introduce a novel evaluation metric to measure the attack’s impact on the original point cloud under the point injection and removal attacks.

## 3. Crafting Adversarial Examples

### 3.1. Problem Formulation

This section presents the formal definition of the LiDAR point cloud segmentation and adversarial example generation mechanisms employed in our study.

In an adversarial attack against LiDAR segmentation, the adversary’s primary goal is to fool the LiDAR segmentation network into assigning the wrong classification label to the LiDAR points by making changes to the point cloud in a way that is imperceptible to human observation but effectively deceives the LiDAR segmentation model. Mathematically, this can be expressed as follows: Let P represent the point cloud which consists of N number of LiDAR points as $P\in R^{N\times 4}$. Each point $P_{i}$ is represented by its 3D coordinates and intensity value as $\left(x_{i},y_{i},z_{i},r_{i}\right)$. The main objective of the semantic segmentation network $M_{seg}$ is to map LiDAR points to labels $y={\left\{y_{i}\right\}}_{i=1}^{N}$, where $y_{i}\in C$ is an element of original class label set $C=\left\{C_{i}|i=1...L\right\}$ with the cardinality of *L* as $M_{seg}\left(P\right)\to y$. The main objective of the attacker is to generate the adversarial point cloud $P_{adv}$ using the adversarial manipulation $m_{adv}$ to obtain the $M_{seg}\left(P_{adv}\right)\to \bar{y}$, where $y\neq \bar{y}$. Specifically, in this study, $P_{adv}$ is crafted using the adversarial manipulations $m_{adv}$, which include point perturbation, point injection, and point removal methods using the knowledge of network gradient information, as discussed in the next sections.

### 3.2. Point Perturbation Attack

Adversarial point perturbation attacks are carried out by slightly changing the coordinates of the points as $\left(x_{i}+\delta_{x},y_{i}+\delta_{y},z_{i}+\delta_{z}\right)$. Specifically, white-box point perturbation attacks are used assuming that the attacker has full access to the network and dataset including original labels obtained via a method such as performing a test step prior to the attack. The most optimal perturbation can be derived by solving a maximization problem given by:

(1) $$\delta^{*}=arg\max_{\delta \in \nabla }L\left(M\left(P+\delta ,\theta \right),y\right).$$

(2) $$L_{dist}\left(P_{org},P_{adv}\right)={∥P_{{org}_{i}}-P_{{adv}_{i}}∥}_{2}^{2}.$$

In Equation (1), L denotes the cost function of the optimization process. The main cost function used in segmentation tasks is Cross-Entropy loss, which calculates the element-wise classification error denoted as $L_{seg}$. In previous research, the imperceptibility of the perturbation attack was regulated by either constraining the perturbation for a specific threshold based on a distance method (norm-bounded attack) or integrating the distance metric, which calculates the difference between the original and corrupted point cloud as a loss function and is iteratively optimized using an optimizer such as Adam [32]. Using the insights from these two approaches, we integrate a distance cost function $L_{dist}$ to the $L_{seg}$ while calculating the gradients for the previous iterative norm-bounded attack sample generation process with the objective of further improving the imperceptibility and stealthiness. Specifically, the L2 loss method is employed as the distance loss, which can be formulated as shown in Equation (2). The generation of point perturbations could be modelled as an optimization process guided by dual loss functions with the objective of maximizing the $L_{seg}$ while minimizing or regulating the distance loss $L_{dist}$. Therefore, the overall loss function of the attack optimization is as shown in Equation (3), where λ is a pre-defined control variable based on the attack’s performance to balance the loss functions.

(3) $$L=L_{seg}-\lambda L_{dist}.$$

In order to generate the adversarial perturbation δ, the previously introduced $l_{\infty }$ norm bounded pixel perturbation attack methods [7,33,34] are used in this study. In particular, the following attack techniques are employed:

**Fast Gradient Sign Method (FGSM)**: FGSM is a single-step attack method and it perturbs the input along the direction of the gradient [7]. The adversarial point cloud from the FGSM attack is given as per the Equation (4). The severity of the perturbation is controlled by the variable ϵ. Specifically, since the FGSM attack is not an iterative attack, the adversarial perturbation is not optimized using the Equation (3). As a result, the preliminary investigations demonstrated a low stealthiness of the attacked samples. To overcome this, as a modification to the original attack perturbations, we clipped and limited the perturbation to the non-negative values.

(4) $$P_{adv}=P+\epsilon .sign\left(\nabla_{P}L\left(M\left(P\right),y\right)\right).$$

**Projected Gradient Descent (PGD)**: PGD attack generates the adversarial inputs by iteratively applying the FGSM attack method with small step size α in *T* amounts of iterations [33]. Generally, the α is set according to ϵ/T≤α≤ϵ. PGD and Basic Iterative Method (BIM) [35] attacks are almost similar, and the only difference is that PGD attack uses a random start for $P^{0}=P+U^{d}\left(-\epsilon ,\epsilon \right)$ where $U^{d}\left(-\epsilon ,\epsilon \right)$ is the uniform distribution between −ϵ and ϵ. The Equation (5) demonstrates the adversarial point cloud from the PGD attack.

(5) $$P_{t+1}^{{}^{\prime }}=clip_{\left(P,\epsilon \right)}\left\{P_{t}^{{}^{\prime }}+\alpha .sign\left(\nabla_{P}L\left(M\left(P_{t}^{{}^{\prime }}\right),y\right)\right)\right\}.$$

**Momentum Iterative Fast Gradient Sign Method (MI-FGSM)**: In this attack method, a momentum term was introduced to the I-FGSM attack method [34]. The main intention behind this momentum term is to introduce transferable adversarial samples by increasing the possibility of reaching the global minimum by escaping the global maxima. This can be mathematically expressed as Equation (6), where the μ and *g* are the decay factor of the momentum and weighted accumulation gradient, respectively. Further, the Equation (7) exhibits the adversarial point cloud from the MI-FGSM attack.

(6) $$g_{t+1}=\mu g_{t}+\frac{\nabla_{P}L\left(P_{t}^{*},y\right)}{{∥\nabla_{P}L\left(P_{t}^{*},y\right)∥}_{1}}.$$

(7) $$P_{t+1}^{{}^{\prime }}=clip_{\left(P,\epsilon \right)}\left\{P_{t}^{{}^{\prime }}+\alpha .sign\left(g_{t+1}\right)\right\}.$$

### 3.3. Point Injection Attack

The point injection attack adds new spoofed points to the most sensitive locations of the given point cloud. Followed by previous studies [30,36,37], a saliency features based point addition and shifting approach is used. The saliency features of each point are calculated using the partial derivative of the loss with respect to each point feature, as shown in Equation (8).

(8) $$S=\{\frac{\partial L_{seg}}{\partial p_{i}}{\}}_{i=1}^{N}.$$

Next, the highest critical points are duplicated based on the saliency scores. Notably, the main loss function utilized in LiDAR segmentation networks is cross-entropy loss and it is essential to have a one-to-one mapping between the number of labels and the number of points available in the network. Hence, the labels of the injected critical points are duplicated in a way similar to the studies [38,39]. Thereafter, the injected points are shifted using the PGD-based point perturbation attack discussed in Section 3.2. The process of point injection attack is defined in Algorithm 1.
**Algorithm 1:** Adversarial point injection attack.
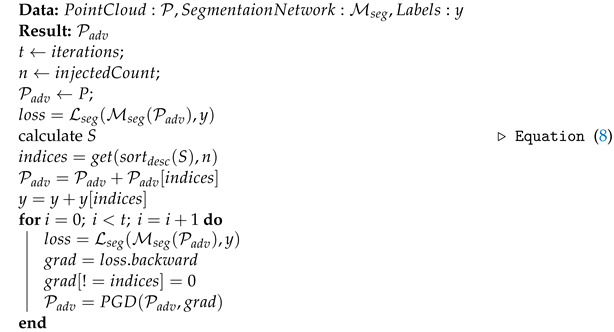


### 3.4. Point Removal Attack

Using the insights gained from the previous studies [30,37], we iteratively remove the *r* percentage of the highest sensitive points from the point cloud. The ratio *r* is a pre-defined variable. As opposed to the point injection attack, when removing the points, the respective label of the point from the original point class label set is deleted. The Algorithm 2 demonstrates the point removal attack.
**Algorithm 2:** Adversarial point removal attack.
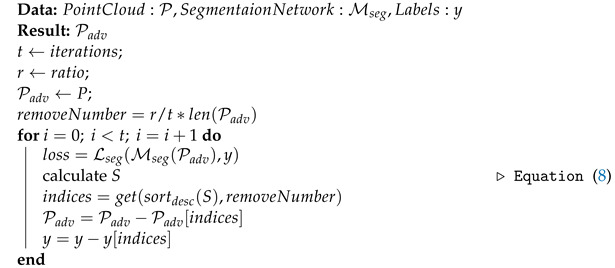


## 4. Evaluation Methods

### 4.1. Robustness Evaluation Metrics

To evaluate the adversarial robustness of the networks under each attack, the robustness score metric $R_{M_{seg}}^{mIOU}$ (Equation (9)) which gives the ratio between mean intersection over union (mIOU) score under clean and attacked samples, is used.

(9) $$R_{M_{seg}}^{mIOU}=\frac{mIOU_{adv}}{mIOU_{clean}}.$$

Moreover, to evaluate the impact on the original point cloud under the point injection attack, we introduce an enhanced version of $R_{M_{seg}}^{\phi }$ named *Robustness Impact Score*$RI_{M_{seg}}^{\phi }$. Here, we first obtain the predictions for the adversarially corrupted point cloud with *K* amount of injected points as $Pred(P_{N+K})$. Then, we remove the predictions of the injected points from the predicted label set and calculate the accuracy. This can be mathematically expressed as Equation (10).

(10) $$RI_{M_{seg}}^{mIOU}=\frac{mIOU_{adv}\left\{Pred\left(P_{N+K}\right)-Pred\left(P_{K}\right)\right\}}{mIOU_{clean}}.$$

When calculating the accuracy or mIOU for the point cloud after the point removal attack, comparing the corresponding ground truth labels without considering the removed points is ineffective because it does not reflect the unavailability of the removed points and its impact on the AV’s perception. As an illustration, suppose a car is on the road and all of its points are removed by an adversary. The accuracy/mIOU for predictions of the remaining points is then calculated by comparing their ground truth labels. However, this method does not effectively quantify the unidentified objects/points due to the point removal attack. Given the importance of this, it is reasonable to interpret these eliminated points as misclassified points. To quantify this phenomenon, a custom label that is not included in the original label set is appended to the removed point indices after receiving the predictions of the corrupted point cloud from the point removal attack and calculating the accuracy and mIOU for $R_{M_{seg}}^{mIOU}$. The mathematical expression for the proposed evaluation method for the point removal attack is shown in Equation (11). Section 6.2 gives an in-depth analysis of these newly proposed metrics for point removal and injection attacks.

(11) $$RI_{M_{seg}}^{mIOU}=\frac{mIOU_{adv}\left\{Pred\left(P_{N-K}\right)+{\left\{Label\right\}}_{i=0}^{K}\right)\}}{mIOU_{clean}}.$$

### 4.2. Attack Imperceptibility Evaluation Metrics

Stealthiness or imperceptibility is an essential feature of adversarially corrupted samples. Hence, the Chamfer Distance (Equation (12)) metric is used to evaluate the difference between original and adversarially corrupted samples.

(12) $$D_{CD}\left(P_{org},P_{adv}\right)=\sum_{x\in P_{org}}\min_{y\in P_{adv}}{∥x-y∥}_{2}^{2}+\sum_{y\in P_{adv}}\min_{x\in P_{org}}{∥x-y∥}_{2}^{2}.$$

Moreover, to benchmark the effectiveness of the proposed dual loss optimization-based perturbation attack method, we propose the metric named change of the Chamfer distance for one unit of mIOU drop as depicted in Equation (13). To be more precise, it gives the difference between original and adversarially corrupted point clouds while degrading the segmentation performance by mIOU 1%.

(13) $$\frac{D_{CD}\left(P_{org},P_{adv}\right)}{mIoU_{clean}-mIOU_{attacked}}.$$

## 5. Experimental Setup

We assess the attack methods against six LiDAR segmentation networks covering three primary data representation techniques namely points, voxels, and point-voxel methods.

As point-based networks, PointNet [40] and PointNet++ [41] networks are used. PointNet architecture consists of three main components, namely: (1) T-Net, which is a spatial transformer to align the point set to canonical space; (2) Multi-Layer Perceptrons (MLP) layers to learn point-wise features, capturing the local characteristics of each point cloud point; and (3) max-pooling layer to learn global features from MLP layers. PointNet learns the features of each point independently. Hence, the structural relationship information between points cannot be captured. As a result, PointNet++, a hierarchical network that extracts features at multiple scales by recursively applying PointNet, was introduced.

This study employs, MinkUnet [42], Cylinder3D [43], and PolarNet [44] networks as the voxel-based networks. MinkUnet is an extension of hierarchical U-Net networks [45] introduced for 2D segmentation. It utilizes novel Minkowski convolutional blocks, which are specifically designed for 3D voxel data. Cylinder3D utilizes a cylindrical representation of voxel space and asymmetrical 3D Convolution kernels to extract features preserving the shape and orientation of objects. PolarNet leverages the strengths of both voxel and BEV representations. Here, the voxel representation is used as the initial input to the network, and then it is transformed into a BEV representation using the polar coordinate system. PolarNet [44] is also based on hierarchical networks and consists of three main components: namely, a feature extractor, a feature aggregator, and a segmentation predictor. Finally, as the point-voxel-based network, we use the SPVCNN [46] network which consists of two branches namely: (1) voxel-based convolutional operation branch which extracts features within individual voxels and incorporates information from neighbouring voxels, and (2) MLP-based point feature extraction branch.

We use the SemanticKITTI dataset [21], which provides 43K LiDAR samples categorized into 23 sequences. In particular, the validation set of the SemanticKITTI dataset is used, as the testing set’s ground truth labels are not publicly available. Notably, evaluating the attack methods against different severity levels on all the 4K LiDAR samples available in the SemanticKITTI validation dataset takes a much longer time. Hence, we use 500 LiDAR samples, which comprise approximately 12% of the validation dataset for faster experiments. For the experiments, we use publicly available code-bases of the networks PointNet: https://github.com/Jiang-Muyun/PointNet12 PolarNet: https://github.com/edwardzhou130/PolarSeg (accessed on 1 July 2023) and mmDetection3D [47] platform. Notably, we train the point-based networks for 360-view LiDAR samples, and for other networks, we use the publicly available checkpoints.

The parameters that remain constant when implementing adversarial attacks are as follows: When evaluating the point injection attacks with different point injection ratios, the point shifting rate ϵ is set as 0.1%, and when evaluating the impact of the point shifting rate we keep point injection ratio as 0.09. Moreover, to perturb the injected points, a PGD attack with $l_{inf}$ norm with 40 iterations is used. In the point removal attack, we set the number of iterations as 10. Finally, in point perturbation attacks (Except FGSM), the number of iterations is set as 40.

## 6. Robustness of Different Segmentation Networks

### 6.1. Evaluating Adversarial Robustness

#### 6.1.1. Adversarial Robustness of Point Perturbation Attacks

**Figure 2:**
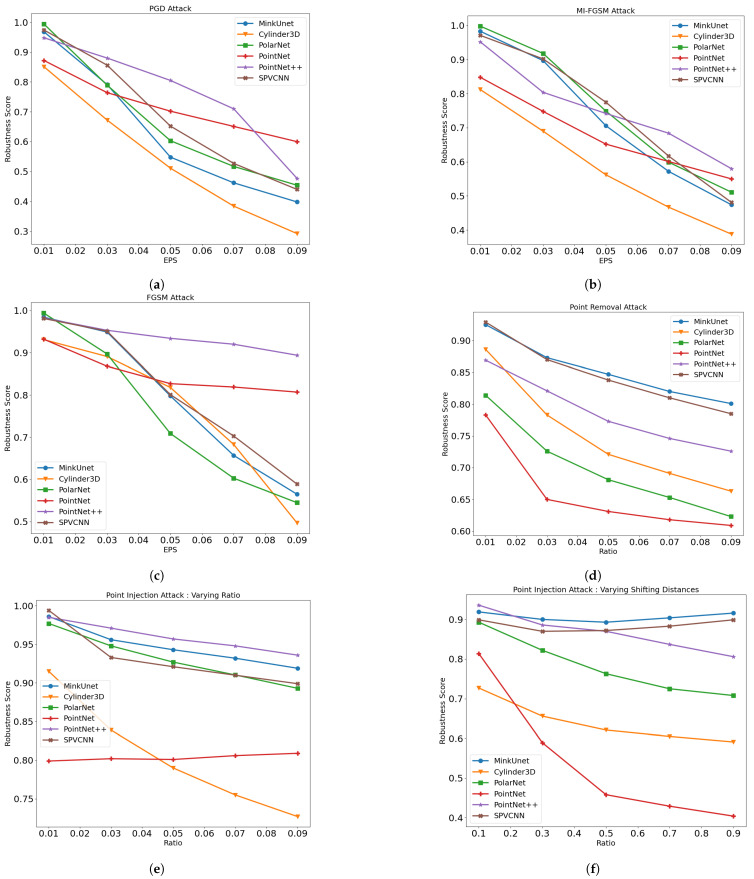
Adversarial robustness of the networks under different severity levels of the attacks. (**a**) Robustness scores under PGD attack with different ϵ values. (**b**) Robustness scores under MI-FGSM attack with different ϵ values. (**c**) Robustness scores under FGSM attack with different ϵ values. (**d**) Robustness scores under point removal attack with different removed ratios. (**e**) Robustness scores under point injection attack with different injection ratios. (**f**) Robustness scores under point injection attack with different ϵ values.

**Figure 3:**
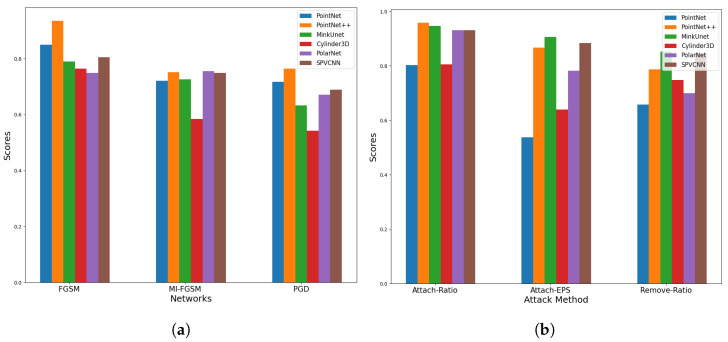
Mean robustness score of the networks against evaluated attack methods. (**a**) Mean robustness score of the Networks against Perturbation Attacks. (**b**) Mean robustness score of the Networks against Point Injection and Removal Attacks.

Table 1 and Figure 2a–c demonstrate the robustness score variation of the different state-of-the-art networks on the SemanticKITTI dataset and Figure 3a depicts the mean robustness score for each point perturbation attack method. These results illustrate that, similar to the image segmentation tasks, iterative attacks are capable of degrading the network’s performance more than the single-step FGSM attack. The examined segmentation networks exhibit similar performance reduction at lower values of ϵ, and when the ϵ value expands, the network’s adversarial robustness degrades significantly.

As per Figure 3a, the Cylinder3D network exhibits the highest susceptibility to perturbation attacks. In contrast, the adversarial vulnerability of two-point-based networks against perturbation attacks is low. Specifically, they demonstrate a higher resilience to the non-iterative FGSM attack method. As we identified, one main reason behind this is that the PointNet and PointNet++ normalize the point coordinates. Hence, the impact of the shifting distances under the perturbation attack is reduced. Further, we notice that the MI-FGSM attack method slightly outperforms the PGD attack approach under the PointNet network. When assessing the attack’s success rate on SPVCNN in contrast to other voxel-based networks, SPVCNN exhibits a notably higher resistance across all three attack methods. One key factor contributing to this resilience is SPVCNN’s use of both voxel and point features and as a result, the network gains a richer understanding of the scenario and stays strong against attacks.

#### 6.1.2. Adversarial Robustness of Point Injection Attacks

We separately analyze the impact of injected point ratio (See Table 2 and Figure 2e) and injected point shifting distance (See Table 3 and Figure 2f) towards the attack’s success rate. These results reveal that the injected point shifting distance has the highest impact over the injected point ratio towards the attack success rate. It can also be observed that the PointNet network is the most vulnerable network while the Cylinder3D network also demonstrates a similar vulnerability. Further, the PointNet network demonstrates nearly constant performance degradation while increasing the injected point ratio. However, when the injected point distance increases, the network demonstrates a significant decrease in resilience. In contrast, the SPCVNN and MinkUnet networks demonstrate a near-constant resilience rate under the varying injected point shifting distances.

#### 6.1.3. Adversarial Robustness of Point Removal Attacks

In Table 4 and Figure 2d, we present the robustness score for the various point removal ratios of the attack using the Equation (11). Similar to the point injection attack, the PointNet network demonstrates the highest susceptibility while MinkUnet demonstrates the highest robustness. In contrast, the Cylinder3D network demonstrates a relatively good performance.

Figure 3 presents the mean robustness scores of the network for each attack method. Based on these scores, we can see that point-based networks are resilient to perturbation attacks. Further, PointNet++ and MinkUnet networks demonstrate a higher resilience against point injection and removal attacks, whereas PointNet demonstrates the least resilience. One could argue that this is because PointNet solely depends on point features and lacks the ability to capture essential information from surrounding points, which may contribute to its increased vulnerability to point injection and removal attacks.

In the next section, we will further discuss the behaviour of each network under the point removal attack and injection attack, comparing the results from the newly proposed equation described in Section 4.1.

### 6.2. Analysis of Updated Robustness Score Methods

**Figure 4:**
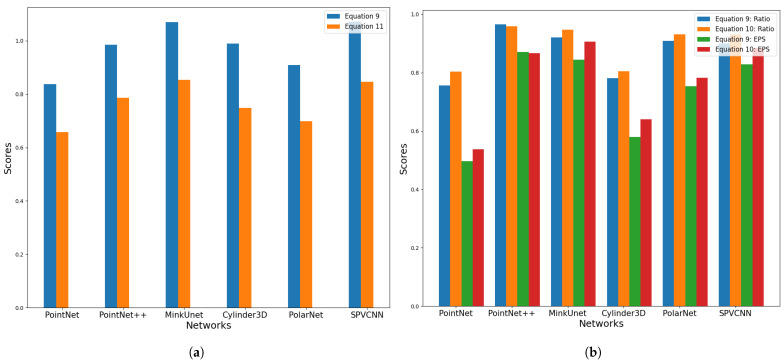
Analysis of Updated Robustness Score Methods for Point Removal and Injection Attacks. (**a**) General robustness score (Equation (9)) metric vs. proposed robustness score metric (Equation (11)): Point Removal Attacks. (**b**) General robustness score (Equation (9)) metric vs. proposed robustness score metric (Equation (10)): Point Injection Attacks.

This section presents a comparison of the robustness score metrics presented in Equations (10) and (11) over Equation (9). From Figure 4a, it is evident that, under PointNet++, MinkUnet, Cylinder3D and SPVCNN networks, Equation (9) gives a robustness score around 1.0, which means the network is able to identify the remaining points after the removal attack correctly and the performance degradation demonstrates by Equation (11) is from the removed points. The main insight gained from this phenomenon is that since LiDAR semantic segmentation is a dense task, removing points from distributed locations cannot have a huge impact on the remaining points.

Figure 4b reveals that the Equation (9) does not correctly reflect the impact of the point injection attack on the original point cloud, as it includes both misclassifications of both original and injected points. Moreover, the outcomes derived from Equation (10) demonstrate that the unlike removing points, injecting points and shifting the distance of injected points has an impact on the predictions of the original points.

### 6.3. Analysis of Class Wise Adversarial Robustness

**Figure 5:**
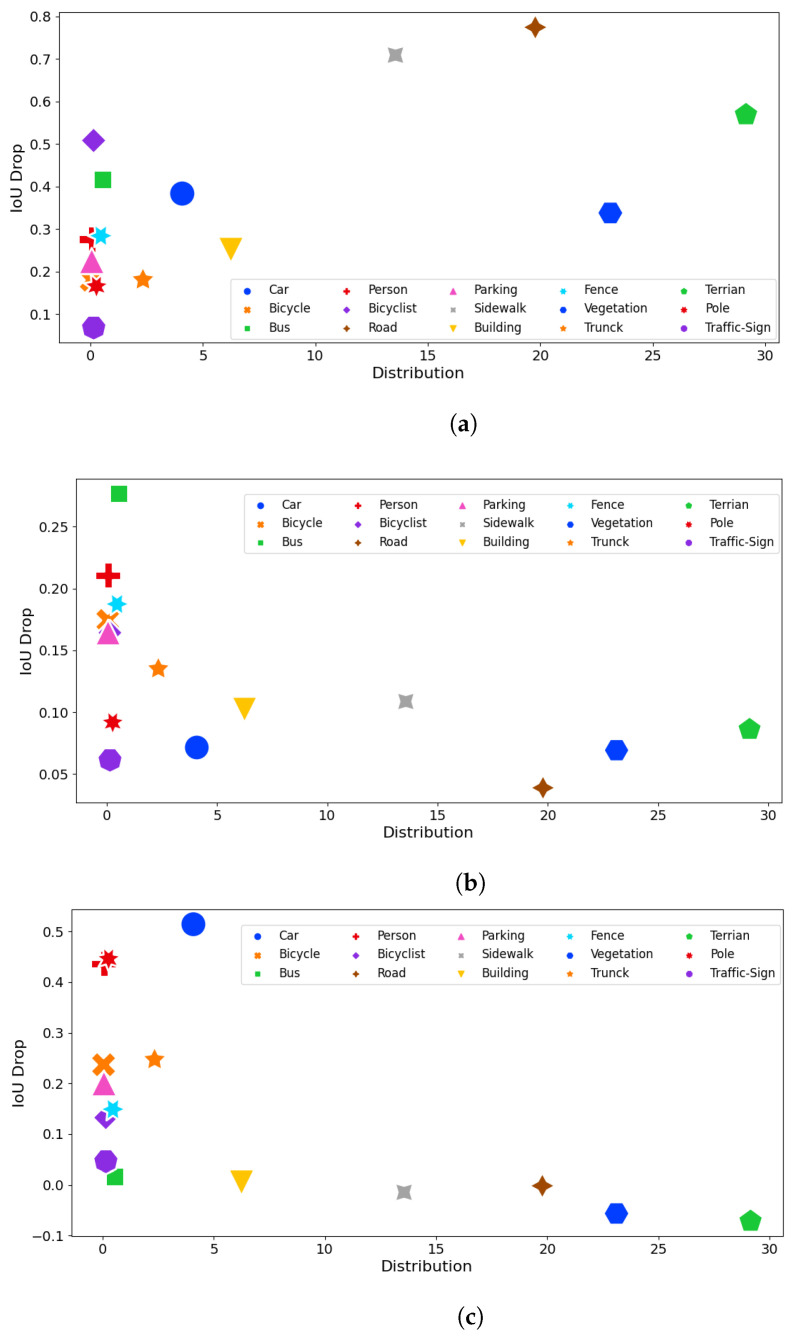
A comparison of class-wise IoU drop when compared to the class-wise point distribution. (**a**) A comparison of class-wise IoU drop when compared to the class distribution under PGD attack. (**b**) A comparison of class-wise IoU drop when compared to the class distribution under Point Injection Attack. (**c**) A comparison of class-wise IoU drop when compared to the class distribution under Point Removal Attack.

In this experiment, we analyzed the class-wise adversarial robustness of each network against the three attack methods. The main intention behind this study is to verify the impact of class-wise point distribution on adversarial attacks and identify the adversarial sensitivity of each class. Notably, we analyze the intersection over union (IoU) difference between (referred to as IoU drop) attacked and corrupted samples using 15 out of 19 classes available in the SemanticKITTI dataset. Figure 5a–c depict the IoU drop of each selected class compared to the available point percentage over the total labeled points (Distribution Ratio).

When considering point perturbation attacks, it is evident that the highly available classes and the classes that reflect ground such as sidewalks, roads, and terrain demonstrate the highest adversarial vulnerability. A notable point that can be seen in point injection and removal attack scenarios is that there is a near-linear relationship between class distribution and IoU drop where the highest available classes are resilient to such attacks. This is because semantic segmentation is a dense task and deleting a relatively small number of points from the highly available classes has no significant impact on it.

## 7. Imperceptibility of the Attack Methods

**Figure 6:**
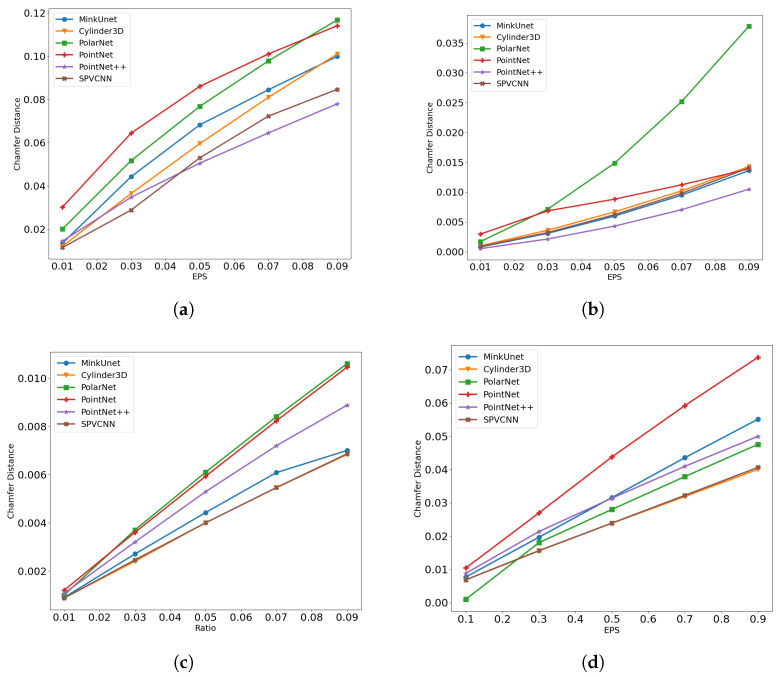
Imperceptibility of the attack methods at different severity levels. (**a**) Attack imperceptibility of the Point Perturbation Attack. (**b**) Attack Imperceptibility of the Point Removal Attack. (**c**) Attack imperceptibility of the Point Injection Attack: varying injection ratios. (**d**) Attack Imperceptibility of the Point Injection Attack: varying shifting levels.

**Figure 7:**
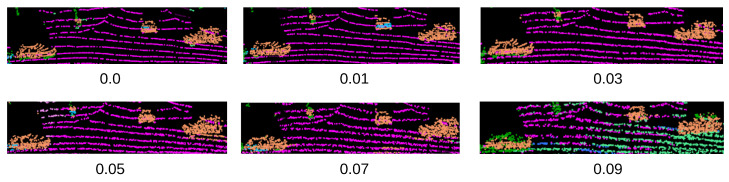
Change to the point cloud which contains cars under the PGD point perturbation attack with different ϵ values.

We evaluate the difference between original and adversarially corrupted LiDAR samples using the Chamfer Distance metric (Equation (12)). Notably, we employ the $l_{2}$ norm-based Chamfer distance approach and report the sum of mean Chamfer distance values from source to target point clouds and vice versa, as implemented in [48]. Figure 6 presents the mean Chamfer Distance of each attack under the various difference severity levels for each network. Moreover, Figure 7 depicts an illustration of a point cloud related to a car under various ϵ values of the PGD attack. Specifically, when it comes to the adversarial point perturbation attacks, Chamfer distances for the PGD attack are presented.

While analyzing the Chamfer distance results, along with the robustness scores presented in Section 6.1, it is possible to observe that point perturbation is the most effective method. However, when it comes to the PointNet network, a point injection attack is effective, as it is able to enable a higher attack success rate while having a high imperceptibility compared to the perturbation attacks. Moreover, the PolarNet network demonstrates an exponential Chamfer distance distribution over the point removal ratios when compared to the other networks.

As mentioned previously, research on the adversarial robustness of 3D point cloud classification and 3D object detection relied on $l_{2}$ norm bounded perturbation attack methods (e.g., -$D_{l2}\left(x+\delta ,x\right)<\epsilon )$ [30,49] or norm-unbounded attack methods [32] with a distance loss function (e.g., -Chamfer Attack [36])-based optimization to regulate the imperceptibility of the attack. However, in this study, we design the adversarial perturbations using both methods. Further, we integrate a distance loss function to the segmentation network loss while calculating the gradients and use those gradients to craft $l_{\infty }$ norm-bounded attacked samples with the intention of further regulating the imperceptibility of the attack method, as discussed in Equation (3). To evaluate the effectiveness of this approach, we conduct a benchmark of the attack methods’ success rate along with their imperceptibility while using our approach and using only the $l_{2}$ norm-bound attack methods using Equation (13). Specifically, the PGD attack with ϵ=0.09 is used for this investigation.

The result presented in Table 5 reveals that our approach is better than just using $l_{2}$ norm-bounded attacks. In addition, these results also reveal the effectiveness of the two point-based networks and the point cloud normalization approach.

## 8. Analysis of Attack Transferability

**Figure 8:**
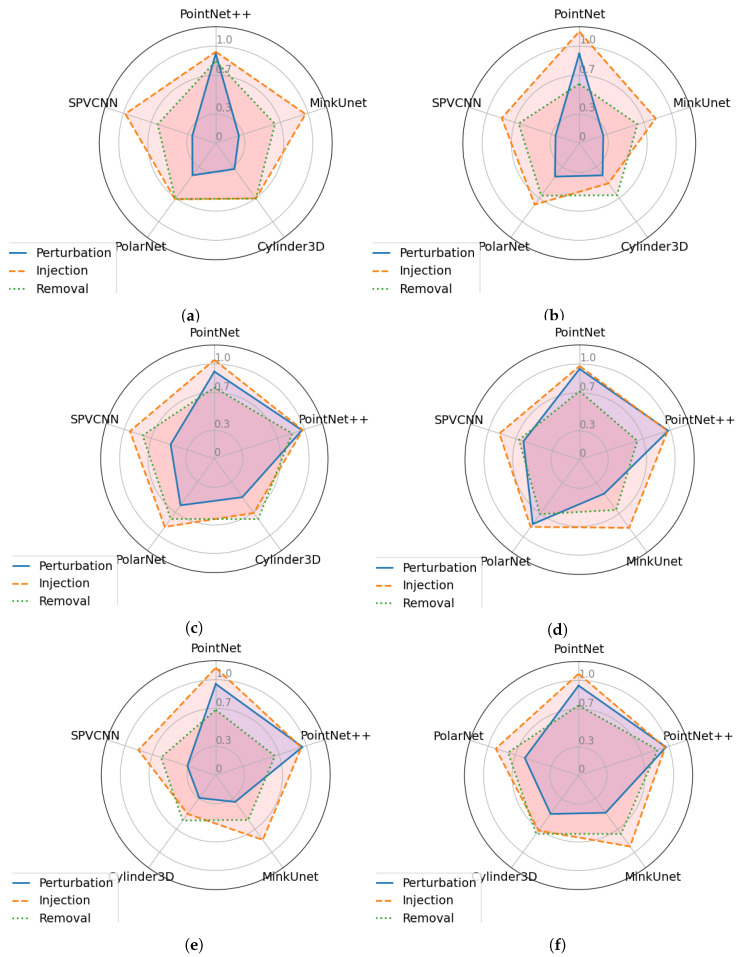
Comparison of Transferability of Attack Samples Generated from each Network. (**a**) Transferability of attacked samples generated from PointNet network. (**b**) Transferability of attacked samples generated from PointNet++ network. (**c**) Transferability of attacked samples generated from MinkUnet network. (**d**) Transferability of attacked samples generated from Cylinder3D network. (**e**) Transferability of attacked samples generated from PolarNet network. (**f**) Transferability of attacked samples generated from SPVCNN network.

This section evaluates the ability of the attack samples produced by one network to deceive the predictions of a different network in a black-box manner. Specifically, we use the PGD attack with ϵ=0.09 as the point perturbation attack, the point injection attack with 0.09 injection ratio, and the PGD-based ϵ=0.9 shifting rate, and finally the point removal attack with 0.09 removal ratio. We present transferability results for the point perturbation attack in Table 6, transferability results for the point injection attack in Table 7, and results for the point removal attacks transferability in Table 8. For better visualization, we present these results in Figure 8.

Based on the data presented in the tables, it is possible to infer that two point-based networks are resistant to attacked samples produced by other networks. Furthermore, when it comes to point-based networks, the point removal and point injection attacks are more effective than the point perturbation attacks. The underlying reason for this phenomenon is that the code base used for PointNet and PointNet++ normalized the coordinates of the points before they were transmitted into the network. As a result, the impact of the point shifting is minimized. Surprisingly, rather than directly performing an attack against a particular network using its gradient information, attacked samples generated from PolarNet and PontNet++ demonstrate a higher attack success rate in most of the evaluations. For example, when adversarially perturbed samples are produced directly from MinkUnet’s gradient information, the resilience score against PGD attack (ϵ=0.09) is 0.398. However, when the LiDAR samples are corrupted using the same PGD attack on PointNet++ and applied to MinkUnet, the robustness score is 0.26. This observation will spark researchers to develop novel black-box attack methods targeting LiDAR perception tasks, employing PointNet and PointNet++ as surrogate networks. Furthermore, it is possible to observe that the Cylinder3D network is highly sensitive to transferable adversarial attack samples, similar to how it is vulnerable to attacks performed directly utilizing its gradient information. In addition, MinkUnet and SPVCNN networks demonstrate similar resilience rates in most of the scenarios.

### Ablation Study on MinkUnet and SPVCNN Networks

**Figure 9:**
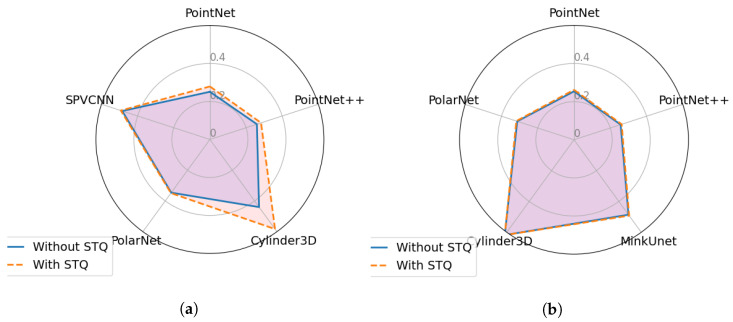
Comparison of transferability of point perturbation attack samples generated from each network with and without using sparse tensor quantization method. (**a**) Robustness scores of MinkUnet network. (**b**) Robustness scores of SPVCNN network.

**Figure 10:**
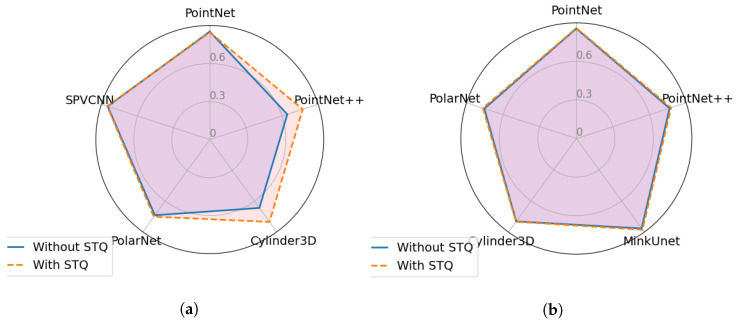
Comparison of transferability of Point Injection Attack samples generated from each network with and without using sparse tensor quantization method. (**a**) Robustness scores of MinkUnet network. (**b**) Robustness scores of SPVCNN network.

Sparse Tensor Quantisation (STQ) is a pre-processing step that is used in the Minkowski Engine [42] which converts the input point cloud into points with distinctive coordinates prior to voxelizing the point cloud. In further detail, this pre-processing step first rounds the coordinates of each point and then keeps only the points with unique coordinates. Both MinkUnet and SPVCNN employ this pre-processing step. However, the results mentioned in the above section for MinkUnet and SPVCNN were achieved without using this method. Hence, this study analyzes the impact of the STQ pre-processing step against point perturbation and injection attacks, as both attack scenarios involve shifting the point coordinates. The radar charts in the Figure 9 and Figure 10 demonstrate the robustness score differences (using Equation (9)) between implementing or not implementing a STQ pre-processing step. Both Figure 9 and Figure 10 illustrate that the STQ method has a minor impact on the robustness against point perturbation and point injection attacks. In particular, only the MinkUnet network demonstrates a slight robustness increment in some attack scenarios while using the STQ method.

## 9. Discussion

In this section, we discuss the key observations of our study based on the formulated research questions. We further discuss the future research directions led by our study.

**RQ1—How robust is LiDAR Semantic Segmentation to adversarial attacks?** Our results reveal that LiDAR semantic segmentation networks are also vulnerable to adversarial attacks. In particular, when comparing the overall results the Cylinder3D network is the most adversarially vulnerable network whereas PointNet++ and MinkUnet demonstrate the highest adversarial resilience. Moreover, the robustness of the SPVCNN network against perturbation attacks, particularly in comparison to voxel-based networks, emphasizes the importance of supplying the network with additional information to enhance its overall resilience. Further, our analysis demonstrates that deleting points from distributed locations has no significant impact on the remaining points. In contrast, the injected points have an impact on the original points.

**RQ2—What are the most adversarially vulnerable classes and what is the impact of class-wise point distribution towards the adversarial robustness?** We demonstrated that point injection and removal attacks have a nearly linear relationship between class-wise point distribution. Further, when it comes to point perturbation attacks, the classes that reflect ground are highly susceptible to adversarial attacks.

**RQ3—How imperceptible are adversarial attacks against LiDAR segmentation?** The Chamfer distance results presented in Section 6 demonstrate that the attack imperceptibility has a relationship with its severity. Moreover, except PointNet network, the point perturbation attack is effective when considering both the attack success rate and the imperceptibility.

**RQ4—How transferable are adversarial attacks on one LiDAR segmentation network to another?** We noticed that, except PointNet and PointNet++, the other networks have a considerable performance degradation for the transfer attack samples. In particular, the attacked samples generated from PointNet and PolarNet demonstrate the highest attack success rate and in several instances, this is better than directly generating the attack samples on a network using its gradient information.

**RQ5—What are the challenges while developing adversarial attacks against LiDAR segmentation?** The first challenge noticed is that similar to the attacks against other point cloud-related tasks, introducing attack methods against LiDAR segmentation is a trade-off between the total attack success rate and the imperceptibility. The next challenge is performing an iterative attack (perturbation, injection, or removal attack) which requires considerably higher computational resources, and these methods are not physically realizable. As a result, the viability of these attacks in real-time is called into doubt. Further, point perturbation attacks mainly altered highly available classes. Hence, targeted attacks may be required to deceive the network into not recognizing other critical classes such as vehicles.

**RQ6—What are the prospective research studies that could be conducted on adversarial robustness of LIDAR segmentation?** It is essential to investigate the adversarial robustness of multi-sensor fusion-based LiDAR segmentation approaches. In the future, it will also be essential to investigate more physically realizable and black-box attack methods against LiDAR segmentation. Moreover, to the best of our knowledge, identifying a training phase attack method against LiDAR segmentation is still an open research problem. Moreover, the adversarial vulnerability of the ground-level points against perturbation attacks enables researchers to develop new attack methods for deceiving steering tasks using techniques such as changing the road surface, etc. In addition, adversarial defense methods against LiDAR segmentation attacks are also a vital topic. Specifically, unlike adversarial training, which enables resilience against only known attack methods, a more generic way of defending against adversarial attacks is essential. Furthermore, adversarial point injection and removal attacks exhibit similar characteristics to common corruptions caused by adverse weather conditions and sensor errors, such as snow, fog, beam missing, and cross-sensor interference, as under these corruptions the point cloud naturally becomes noisy or sparse [50]. As a potential solution, in the future, we plan to investigate and develop a point cloud reconstruction network based on generative networks to mitigate both man-made adversarial attacks and common corruptions.

## 10. Conclusions

The adversarial robustness of AVs is a vital field of research. Previous studies on adversarial attacks against AV perception tasks mainly focused on 2D image-based approaches and 3D object detection. However, the adversarial vulnerability of LiDAR segmentation is a relatively unexplored topic. Hence, this paper presents an extensive analysis of the adversarial robustness of 3D LiDAR semantic segmentation using the SemanticKITTI dataset. In particular, we systematically investigate different LiDAR semantic segmentation networks spanning three data representation strategies and three different attack methods. We then evaluate the transferability and imperceptibility of these attack methods. After analyzing the results, we present numerous observations for future research and challenges of developing attacks against LiDAR segmentation. As a limitation, our study does not assess the adversarial robustness of range-image-based LiDAR segmentation networks. We hope our study will enable valuable insights for future research to improve the adversarial robustness of LiDAR semantic segmentation in autonomous vehicles.

## Figures and Tables

**Table 1:** Networks’ robustness score against different point perturbation attacks.

Attack	Network	${\mathrm{mRI}}_{M_{\mathrm{seg}}}^{\mathrm{mIOU}}↑$	ϵ: 0.01	ϵ: 0.03	ϵ: 0.05	ϵ: 0.07	ϵ: 0.09
	PointNet	0.850	0.932	0.868	0.827	0.819	0.807
	PointNet++	0.936	0.982	0.953	0.934	0.920	0.894
FGSM	MinkUnet	0.790	0.984	0.949	0.798	0.657	0.565
	Cylinder3D	0.764	0.931	0.891	0.818	0.683	0.497
	PolarNet	0.749	0.994	0.897	0.709	0.603	0.545
	SPVCNN	0.805	0.981	0.951	0.801	0.703	0.589
	PointNet	0.681	0.848	0.748	0.652	0.601	0.559
	PointNet++	0.752	0.952	0.804	0.742	0.684	0.580
MI-FGSM	MinkUnet	0.726	0.983	0.897	0.706	0.572	0.474
	Cylinder3D	0.585	0.812	0.690	0.562	0.467	0.388
	PolarNet	0.755	0.998	0.918	0.749	0.599	0.511
	SPVCNN	0.749	0.971	0.902	0.775	0.617	0.481
	PointNet	0.717	0.872	0.764	0.702	0.651	0.600
	PointNet++	0.764	0.948	0.880	0.805	0.710	0.477
PGD	MinkUnet	0.633	0.968	0.791	0.548	0.462	0.398
	Cylinder3D	0.542	0.851	0.672	0.511	0.384	0.292
	PolarNet	0.671	0.994	0.789	0.603	0.517	0.454
	SPVCNN	0.689	0.974	0.856	0.652	0.527	0.440

**Table 2:** Networks’ robustness score against point injection attack under different injected point ratios.

Network	${\mathrm{mRI}}_{M_{\mathrm{seg}}}^{\mathrm{mIOU}}↑$	*r*: 0.01	*r*: 0.03	*r*: 0.05	*r*: 0.07	*r*: 0.09
PointNet	0.803	0.799	0.802	0.801	0.806	0.809
PointNet++	0.959	0.985	0.971	0.957	0.948	0.936
MinkUnet	0.947	0.986	0.956	0.943	0.932	0.919
Cylinder3D	0.805	0.915	0.839	0.790	0.755	0.727
PolarNet	0.931	0.977	0.948	0.927	0.910	0.893
SPVCNN	0.931	0.994	0.933	0.921	0.910	0.899

**Table 3:** Networks’ robustness score against point injection attack under different shifting values.

Network	${\mathrm{mRI}}_{M_{\mathrm{seg}}}^{\mathrm{mIOU}}↑$	ϵ: 0.1	ϵ: 0.3	ϵ: 0.5	ϵ: 0.7	ϵ: 0.9
PointNet	0.538	0.814	0.589	0.458	0.429	0.404
PointNet++	0.867	0.936	0.886	0.870	0.837	0.806
MinkUnet	0.906	0.919	0.900	0.893	0.904	0.916
Cylinder3D	0.640	0.727	0.656	0.621	0.605	0.591
PolarNet	0.782	0.893	0.822	0.763	0.725	0.708
SPVCNN	0.884	0.899	0.870	0.872	0.883	0.899

**Table 4:** Networks’ robustness score against point removal attack under different removed point ratios.

Network	${\mathrm{mRI}}_{M_{\mathrm{seg}}}^{\mathrm{mIOU}}↑$	*r*: 0.01	*r*: 0.03	*r*: 0.05	*r*: 0.07	*r*: 0.09
PointNet	0.658	0.783	0.650	0.631	0.618	0.609
PointNet++	0.787	0.869	0.821	0.773	0.746	0.726
MinkUnet	0.853	0.925	0.873	0.847	0.820	0.801
Cylinder3D	0.748	0.886	0.783	0.721	0.691	0.663
PolarNet	0.699	0.814	0.726	0.681	0.653	0.623
SPVCNN	0.846	0.929	0.870	0.838	0.810	0.785

**Table 5:** Benchmark of the attack’s effectiveness while using $l_{2}$ norm-bounded attacks and using our approach. Note: lower is better.

Network	PointNet	PointNet++	MinkUnet	Cylinder3D	PolarNet	SPVCNN
$l_{2}$ Attack	4.25	1.97	0.34	0.274	0.483	0.316
Ours	2.37	0.78	0.282	0.255	0.414	0.253

**Table 6:** Transferability of the Point Perturbation Attack. Note:- Net-G: Network that is used to generate attacked samples. Net-A: the network that is used for the evaluations.

	Net-G	PointNet	PointNet++	MinkUnet	Cylinder3D	PolarNet	SPVCNN
Net-A							
PointNet		-	0.923	0.920	0.949	0.956	0.946
PointNet++		0.924	-	0.973	0.977	0.957	0.968
MinkUnet		0.251	0.260	-	0.440	0.346	0.485
Cylinder3D		0.328	0.409	0.502	-	0.295	0.500
PolarNet		0.408	0.425	0.608	0.834	-	0.595
SPVCNN		0.254	0.257	0.486	0.618	0.313	-

**Table 7:** Transferability of the Point Injection Attack. Note:- Net-G: Network that is used to generate attacked samples. Net-A: the network that is used for the evaluations.

	Net-G	PointNet	PointNet++	MinkUnet	Cylinder3D	PolarNet	SPVCNN
Net-A							
PointNet		-	1.152	1.043	0.979	1.126	1.07
PointNet++		0.943	-	0.981	0.970	0.944	0.958
MinkUnet		0.973	0.828	-	0.883	0.839	0.923
Cylinder3D		0.703	0.511	0.708	-	0.502	0.716
PolarNet		0.714	0.782	0.890	0.871	-	0.916
SPVCNN		0.980	0.840	0.938	0.883	0.853	-

**Table 8:** Transferability of the Point Removal Attack. Note:- Net-G: Network that is used to generate attacked samples. Net-A: the network that is used for the evaluations.

	Net-G	PointNet	PointNet++	MinkUnet	Cylinder3D	PolarNet	SPVCNN
Net-A							
PointNet		-	0.611	0.751	0.716	0.684	0.737
PointNet++		0.847	-	0.863	0.636	0.652	0.874
MinkUnet		0.642	0.631	-	0.652	0.580	0.760
Cylinder3D		0.704	0.662	0.788	-	0.588	0.759
PolarNet		0.710	0.667	0.786	0.705	-	0.780
SPVCNN		0.630	0.657	0.796	0.667	0.603	-

## Data Availability

Used publicly available data from http://www.semantic-kitti.org/ (accessed on 1 July 2023).
